# Awareness and Acceptance of Hematopoietic Stem Cell Transplantation for Sickle Cell Disease in Jazan Province, Saudi Arabia

**DOI:** 10.7759/cureus.21013

**Published:** 2022-01-07

**Authors:** Eman Hurissi, Alaa Hakami, Jawahir Homadi, Fatimah Kariri, Ethar Abu-Jabir, Rahaf Alamer, Raghad Mobaraki, Abdulaziz A Jaly, Edrous َAlamer, Abdulaziz H Alhazmi

**Affiliations:** 1 Medicine and Surgery, Jazan University, Jazan, SAU; 2 Pharmacy, Jazan University Hospital, Jazan, SAU; 3 Department of Medical Laboratories Technology, College of Applied Medical Sciences, Jazan University, Jazan, SAU; 4 Microbiology, Jazan University, Jazan, SAU; 5 Medical Research Center, Jazan University, Jazan, SAU

**Keywords:** sickle cell disease (scd), kingdom of saudi arabia (ksa), jazan city, hematopoietic stem cell transplant, clinical hematology

## Abstract

Background: Sickle cell diseases (SCD) are a group of inherited disorders that lead to abnormal beta subunits of hemoglobin (Hb) and are characterized by several complications which can be life-threatening. The prevalence of this disease is high in Jazan province, Saudi Arabia. The current protocol for the treatment of SCD is mainly based on alleviating signs and symptoms to avoid severe complications. Hematopoietic stem cell transplant (HSCT) is considered a definitive therapy for SCD. However, there is a long patient waiting list for HSCT in Saudi Arabia. A lack of community awareness and incorrect information about the importance of HSCT in SCD treatment is believed to be contributing to the shortage in HSCT. Thus, this study aims to assess community awareness and attitudes toward HSCT as a cure for SCD in Jazan province, Saudi Arabia.

Methods: An observational cross-sectional retrospective study was conducted in Jazan province. General and demographic data were collected and pretested survey including questions about public knowledge and attitude toward HSCT for SCD were answered. Both t-test and chi-square tests were used for analysis.

Results: 1167 participants were included in this study with a mean age of 26 (SD: 8). About 50% of the study participants believed that SCD can be treated and 78% of the study participants already have heard about HSCT. About 57% of the participants defined HSCT correctly and 42% were willing to donate. Better knowledge and positive attitude toward HSCT were significantly reported among patients with SCD or their relatives as well as among people with higher education and healthcare workers (HCW).

Conclusion: About 57% of the study participants were able to define HSCT and most related questions were answered correctly. A positive correlation was found between the knowledge about HSCT and people with higher education or those who were diagnosed with SCD or their relatives and friends. Further, only 42% of our study participants were willing to donate, a percent that is positively associated with better knowledge about HSCT. National education programs are needed to enhance the overall awareness of Jazan communities toward HSCT which could contribute to reducing the number of patients waiting for HSCT.

## Introduction

Sickle cell diseases (SCD) are a group of inherited disorders that affect the beta subunits of hemoglobin (Hb). This disease is characterized by the presence of at least one copy of Hb “S”. The disease can be homogeneous as SS phenotype or heterogeneous as SC. This pathogenic Hb can result in abnormality in Hb polymerization which may result in abnormality in the morphology of red blood cells (RBCs) [1]. Individuals carrying this abnormal form of Hb usually experience painful episodes which are known as vaso-occlusive crises (VOC). These crises are mediated by the occlusion of small blood vessels with abnormally shaped RBCs [1].

Globally, the prevalence of SCD is almost 112 per 100 000 [2]. SCD is more commonly reported in sub-Saharan African countries, Saudi Arabia, India, south and central America, and the Mediterranean countries [1]. Among all middle east countries, Saudi Arabia has been reporting the highest prevalence of SCD cases. Most of these cases were reported from the eastern part of the country, followed by the southwestern region of Saudi Arabia [3]. Jazan province is located in the southwestern region of Saudi Arabia and is considered one of the highest prevalent regions for SCD (reporting about 3%) [3]. A recent study among patients with SCD in Jazan province showed that VOC was reported as the most common cause of hospital admission [4].

The current treatment protocol for SCD is limited to the management of the clinical manifestations which includes the use of hydroxyurea, blood transfusions, and other supportive care such as analgesic, prophylactic antibiotic prescription, and psychological support, which have improved the life expectancy of patients with SCD [5]. Hydroxyurea is considered an effective drug to reduce the frequency of painful crises [6]. Blood transfusion has its known clinical indication in patients with SCD such as aplastic, hemolytic, and sequestration crises. Furthermore, the protocols of chronic blood transfusion are implemented in selective cases such as acute chest syndrome (ACS), priapism, pulmonary hypertension, renal dysfunction, and hepatopathy [7]. These supportive treatments do not prevent SCD-mediated organ injury, which accounts for almost 14% mortality rate in children and adults [8]. Furthermore, adults with SCD may experience consistent disability, pain, and poor quality of life [9]. In the previous years, HSCT has been shown as the only curative treatment for SCD [10].

Generally, the knowledge and acceptance of the HSCT are low worldwide [11], and overall there is a long waiting list for HSCT. It is estimated that almost 60% and 30% of Saudi children and adult patients, respectively, who seek HSCT do not find well-matched donors [12]. A major reason for the inadequacy in the number of available HSC donors has been attributed to the lack of community awareness and the availability of incorrect information about the importance of HSCT in SCD treatment [13]. Data on the community awareness and attitudes toward HSCT in Saudi Arabia are limited. Thus, in this study, we aim to assess the overall awareness and acceptance of HSCT for the treatment of sickle cell anemia among the Jazan population.

## Materials and methods

Study subjects and design

We conducted this study using a cross-sectional observational design, using an online survey that targeted adults in Jazan province. Jazan is located in the southwest corner of Saudi Arabia which harbors almost 2 million inhabitants [14]. The survey was distributed based on data collectors and investigators’ networks. The questionnaire was designed after going through previous articles with the same topic and asking an expert in the field [11,13,15]. The internal consistency and external reliability of the survey were evaluated. Internal consistency was done using Cronbach’s alpha and resulted in values over 0.80. The external reliability was tested using test-retest with a score over 0.80. Further, before the distribution of the questionnaire, a pilot sample (n = 20) was used to evaluate the clarity and wording of the questionnaire items. Of note, answers from this pilot sample were excluded from the final analysis. The questionnaire started with questions to collect demographics such as age, gender, current education level, parents’ monthly income, socioeconomic status, and the level of participants’ education. Following this, participants were asked general questions about HSCT as a treatment for SCD (summarized in Table 1). The study excluded responses that did not contain informed consent, data for participants who provided incomplete responses, and responses of individuals from outside of Jazan province. Data were primarily collected during the first 2 weeks of September 2021.

Ethical approval

Ethical approval for conducting this study was obtained from the ethical approval committee at Jazan University (reference number; REC42/1/087, date 22 March 2021). Consents were taken from all participants prior to participation in the study. The study excluded all participants who refused to participate or those who were outside Jazan province, Saudi Arabia.

Sample size and statistical analysis

The sample size for this study was calculated using the Raosoft sample size calculator (Raosoft Inc., Seattle, WA, USA, www.raosoft.com). As the population of Jazan province is estimated to be around 2 million inhabitants [14], we determined that 384 participants were enough to reach a 95% CI and 5% margin of error. However, to reduce sampling bias in our method as this study was based on an online questionnaire distributed via social media, we increased the sample size to include 1167 participants, which is more than threefolds of the required size. Descriptive statistics were performed for the collected data. Both the chi-square and t-test were performed using SPSS v.23 (IBM Corp., Armonk, NY, USA) with the alpha criterion for p-value set at 0.05.

## Results

Sociodemographic characteristics of the study subjects

This questionnaire was completed by a total of 1167 participants who met our inclusion criteria, of whom 43% were male with a mean age of 26 years (SD: 8). Almost 7% of the study participants had SCD and 3% were the parents of patients with this disease. Participants who were relatives or had friends with SCD represented 26% or 28% of the total study participants, respectively. Most of the study participants were students (38%), and about 31% of the participants were employed (21% were not healthcare workers (HCWs) and 10% reported to be HCWs), while 17% of the study participants were unemployed. A large number of the study participants were educated with 75% of them having university levels of education. Participants were then asked whether they believe that SCD can be cured. In response to this, around half of the participants (48%) reported that SCD is curable. Participants were also followed up by questioning them if they knew about HSCT. Participants’ responses showed that almost 78% of the participants seemed to have heard about HSCT in the past. These results are summarized in Table 1.

**Table 1:** General characteristics of the participants in our study SD: standard deviation; SCD: sickle cell disease; HCW: healthcare worker; SAR: Saudi Riyals; HSCT: hematopoietic stem cell transplantation

Variable	Participants, n= 1167
Age, years (mean; SD)	26; 8
Sex, n (%)	
Male	501 (43%)
Female	1117 (57%)
Classification of participants: n (%)	
Patient with SCD	78 (7%)
Parents of patients with SCD	33 (3%)
Relative of patients with SCD	305 (26%)
Friend of patients with SCD	326 (28%)
I do not know any patient with SCD	425 (36%)
Occupation, n (%)	
HCW	118 (10%)
Non-HCW	238 (21%)
Student	444 (38%)
Freelancer	60 (5%)
Housewife	108 (9%)
Looking for job	199 (17%)
Marital status, n (%)	
Single	648 (55%)
Married	491 (42%)
Divorced	20 (2%)
Widow	8 (1%)
Highest education, n (%)	
Uneducated	7 (1%)
High school	244 (21%)
University level	879 (75%)
Postgraduate level	37 (3%)
SCD can be treated? n (%)	
No	566 (22%)
Yes	257 (48%)
I do not know	344 (30%)
Heard about HSCT before? n (%)	
Yes	912 (78%)
No	255 (22%)

Knowledge and attitude toward HSCT

The survey further explored the overall knowledge and attitude of the participants toward HSCT through questioning them multiple questions related to HSCT (Table 2). About 57% of the study participants correctly defined HSCT as “stem cells collected from suitable donors are injected into the recipient’s body.” Analysis of the responses showed that few participants had heard about HSCT in the past although they did not correctly define it (10%) and almost a third of the study participants knew nothing about HSCT. Only 14% of the study participants thought that HSCT could induce severe complications with 44% and 42% of the participants believing in no subsequent complication and “they did not know about the HSCT consequences,” respectively. Donation or acceptance of HSCT by participants or their children were reported by more than half of the study participants (58%) (Table 2).

**Table 2:** Knowledge about HSCT HSCT: hematopoietic stem cell transplantation; Hb: hemoglobin; SA: Saudi Arabia

Variable	Participants, n= 1167
What do you know about HSCT? n (%)	
Nothing	382 (33%)
A blood transfusion process	37 (3%)
A surgery where bones are cut and given to another person	85 (7%)
Cells collected from suitable donors Injected into the recipient’s body	663 (57%)
HSCT results in severe complications? n (%)	
Yes	162 (14%)
No	520 (44%)
I do not know	485 (42%)
Do you know someone who has undergone HSCT?, n (%)	
Yes	279 (24%)
No	888 (76%)
Would you like to donate bone marrow or undergo a bone marrow transplant, or allow your child to receive or donate? n (%)	
Yes	487 (42%)
No	680 (58%)

Based on participants’ responses on hearing or not about HSCT in the past, we categorized our study participants into two main groups, i.e., participants who were not aware of HSCT and those who were aware of HSCT (Table 3). These two groups were then compared against each other using several parameters including age, gender, classification of participants (SCD patients, parents of SCD patients, relatives or friends of SCD patients), education levels, employment status, and if they knew of individuals who received HSCT in the past. As shown in Table 3, the chi-square test and t-test for univariate analysis showed that several factors have significant associations with HSCT awareness. These factors include gender (p = 0.010), being either SCD patients, parents, relatives or friends of SCD patients (p = 0.0001), employment status (p = 0.015), and level of education (p = 0.0001). Further, we grouped the study participants into two main groups based on their willingness to donate HSCT or not, then we tested different parameters among the two groups to identify which parameters that may mediate such willingness. The parameters tested included the abovementioned parameters (parameters used in Table 2) in addition to asking participants more questions about the potential complications following HSCT (Table 3).

**Table 3:** Comparison between participants who have heard about HSCT against those who have not heard about HSCT SD: standard deviation; SCD: sickle cell disease; HCW: healthcare worker; SAR: Saudi Riyals; HSCT: hematopoietic stem cell transplantation. #The alpha criterion for p-value was set to 0.05. *Significant in univariate analysis.

Variable	Not Aware about HSCT= 255 (22%)	Aware about HSCT= 912 (78%)	p-value#
Age, years (mean; SD)	27.6; 8	28.4; 8	0.204
Sex, n (%)			0.010*
Male	128(50%)	373(41%)	
Female	127 (50%)	539(59%)	
Classification of participants: n (%)			0.0001*
Patient with SCD	13 (5%)	65 (7%)	
Parents of patients with SCD	3 (1%)	30 (4%)	
Relative of patients with SCD	56 (22%)	249 (27%)	
Friend of patients with SCD	58(23%)	268 (29%)	
I do not know any patient with SCD	125 (49%)	300 (33%)	
Occupation, n (%)			0.015*
HCW	14 (5%)	104 (11%)	
Non-HCW	60 (24%)	178 (19%)	
Student	88 (34%)	356 (39%)	
Freelancer	18 (7%)	42 (5%)	
Housewife	30 (12%)	78 (8%)	
Looking for job	45 (18%)	154 (18%)	
Education, n(%)			0.0001*
Uneducated	3 (1%)	4 (1%)	
High school	73 (29%)	171 (19%)	
University level	178 (69%)	701 (77%)	
Postgraduate level	1 (1%)	36 (3%)	

Data analysis showed that being a patient with SCD, parents of SCD patients, relatives, or friends of SCD patients enhanced positive attitude toward HSC donation among our study participants. Several other factors also had positive associations with the inclination toward HSC donation including employment status (p = 0.003), overall knowledge on SCD and HSCT; if they knew that SCD is treatable (p = 0.0001), potential HSC complication (p = 0.0001) or having known someone who has undergone HSCT (p = 0.0001) (Table 4).

**Table 4:** Comparison between participants who are willing to donate and who are not willing SD: standard deviation; SCD: sickle cell disease; HCW: healthcare worker; SAR: Saudi Riyals; HSCT: hematopoietic stem cell transplantation; SA: Saudi Arabia #The alpha criterion for p-value was set to 0.05. *Significant in univariate analysis.

Variable	Willing to donate HSC = 487 (42%)	Not willing to donate HSC = 680 (58%)	p-value #
Age, years (mean; SD)	28: 8	28: 8	0.398
Sex, n (%)			0.402
Male	285 (59%)	381 (56%)	
Female	202 (41%)	299 (44%)	
Are you, n (%)			0.0001*
Patient with SCD	49 (10%)	29 (4%)	
Parents of patient with SCD	22 (5%)	11 (2%)	
Relative of patient with SCD	140 (29%)	165 (24%)	
Friend of patient with SCD	139 (29%)	187 (28%)	
I do not know any patient with SCD	137 (28%)	288 (42%)	
Occupation, n (%)			0.003*
HCW	61 (13%)	57 (8%)	
Non-HCW	77 (16%)	161 (24%)	
Student	185 (38%)	259 (38%)	
Freelancer	32 (7%)	28 (4%)	
Housewife	43 (9%)	65 (10%)	
Looking for job	89 (18%)	110 (16%)	
Highest education, n(%)			0.071
Uneducated	2 (1%)	5 (1%)	
High school	100 (20%)	144 (21%)	
University level	362 (72%)	517 (76%)	
Postgraduate level	23 (5%)	14 (2%)	
Do you think those who have undergone HSCT will complain from the complication? n (%)			0.0001*
No	279 (57%)	241 (35%)	
Yes	60 (12%)	102 (15%)	
I do not know	148 (30%)	337 (50%)	
Do you know someone who has undergone HSCT? n (%)			0.0001*
No	324 (67%)	564 (83%)	
Yes	163 (33%)	116 (17%)	
Do you have an idea of how much HSCT cost in SA? n (%)			0.0001*
It costs a lot of money	54 (11%)	62 (9%)	
It’s affordable	4 (1%)	2 (0%)	
I have no idea	268 (55%)	498 (73%)	
Free	161 (33%)	118 (17%)	

## Discussion

SCD is a genetic condition that is characterized by a short lifespan, crescent-shaped RBCs which is mediated by the existence of abnormal Hb. This abnormality is pathogenic to different organs and may cause multiple complications such as VOC, ACS, and repeated infections [1]. Consequently, this disease requires a frequent need for health services for screening, vaccination, medications, blood transfusions, and hospitalizations to reduce the potential associated morbidity and mortality [16]. Jazan province is considered one of the highest prevalent regions for SCD in Saudi Arabia. A study conducted by Alsaeed and his group in 2018 reported about 7% prevalence of SCD among the Jazan population where VOC and ACS were the major causes for hospital admission, accounting for 56% and 12%, respectively [17]. Saudi Arabia has one of the world’s largest series for HSCT [18]. A total of 6184 HSCTs were conducted in Saudi Arabia between 1984 and 2016 which were mainly performed to treat malignancies such as acute lymphoblastic leukemia, acute myelogenous leukemia, and chronic myelocytic leukemia. HSCT is the most effective therapy for life-threatening blood disorders, including SCD and others [8]. Despite the high prevalence rate of SCD in Jazan, there are limited studies in Saudi Arabia or Jazan province to evaluate public awareness, knowledge, and attitude toward HSCT. Although few studies were conducted previously to evaluate the overall awareness and attitude toward HSCT in different provinces in Saudi Arabia, including Jazan, and these studies targeted a certain group of individuals (students of medical and biomedical background). A study carried out in 2016 in Riyadh, Saudi Arabia found that the level of knowledge among nursing students was poor, and the knowledge was enhanced by educational programs [19]. Moreover, Hazzazi et al. conducted a study in 2019 to measure the awareness of medical students in Jazan University about HSCT and they found that the majority of the medical students lacked the proper knowledge on HSCT and only about 9% of the participants were willing to donate [20]. Moreover, in Taif, Saudi Arabia, another study was published in 2020 which aimed to measure the knowledge and attitude of medical students of Taif university toward HSCT [15]. The study showed a low level of basic knowledge about HSC and only 2% of the participants donated bone marrow in the past [8]. Another study that targeted the public population in Saudi Arabia concluded that the overall knowledge of the study subjects about HSCT was low, and a positive correlation was found between the level of education and the knowledge about HSCT [15]. Therefore, in this study, we have conducted a cross-sectional, observational study to assess the overall awareness and attitudes of adults on HSCT, in Jazan province, Saudi Arabia.

In the current study, which targeted the general population, we found that almost 78% of the study participants already had heard about HSCT in the past (Table 1), and 57% defined HSCT correctly (Table 2). Moreover, a better level of knowledge and attitude was associated with people with higher education or individuals with medical professionals (57%) compared to the previously reported studies which targeted medical students [15,19,20]. This difference in knowledge level could be explained as the current study enrolled HCWs (10%) that usually face patients with SCD as a regular practice and knew that HSCT is considered a potential treatment for SCD. Further, when it comes to students, our data are in line with previous reports as we found no significant difference neither in knowledge nor in attitude toward HSCT (Tables 3-4). In the current study, we also found that the female gender is significantly associated with better knowledge; however, a difference in positive attitude toward HSCT was not affected by gender differences (Tables 3-4). This finding is similar to another study that was conducted in Riyadh [21], which was conducted on the general population. The authors reported that being female was significantly associated with better knowledge about HSCT [21]. This finding could be more related to our study population, in which we had an over-representation of females compared to males (57% vs 43%). Further, in our region, it is known that mothers or sisters of patients with SCD usually accompany their patients during hospital admission, and that may lead to some extent of exposure to HSCT as a cure for SCD. In Africa, Adediran et al. conducted a study in a tertiary hospital in Nigeria in 2016 and found that about 65% of the participants were aware of HSCT and the level of knowledge was significantly associated with better attitude [11]. It seems explainable that the difference between the percent of knowledge in our study (57%) and what Adediran et al. reported in Nigeria (65%) could be related to the type of individuals recruited in both studies, i.e., our study targeted the general public, while Nigerian study included individuals from hospital settings [11]. Thus, we found in this study a better level of knowledge and attitude about HSCT among patients with SCD and their relatives and friends compared to those who did not know any SCD patients (Tables 3-4). Another report from Malaysia conducted among nursing students found a poor correlation between knowledge and attitude toward HSCT. In our study, only 47% of those who have heard about HSCT were willing to donate, despite 70% of them being able to define HSCT correctly. This correlation between the knowledge and the attitude indicates that the knowledge alone is not sufficient to display a good attitude toward HSCT and probably other cultural factors may exist which could play essential roles in that [13].

In Saudi Arabia, HSCT is free and there is a real need for HSCT donors [12,22]; however, only 24% of our participants knew this fact (Table 2) and this proportion is positively associated with better knowledge and attitude toward HSCT (Tables 3-4). Thus, huge efforts are warranted by health officials to conduct campaigns to target medical and public communities, an action that may improve awareness and acceptance of HSCT [13,23]. This level of awareness may be ameliorated at medical school and public levels. In Mayo clinic, USA, a team was able to identify knowledge gaps regarding the donation process and HSCT knowledge [24]. It was suggested that multicenter multi-subject programs may better assess the role of medical education in increasing awareness and attitude toward HSCT [24]. Thus, similar programs are warranted at the national level and could be integrated into the curriculum or extracurricular activities of medical schools in Saudi Arabia. To increase awareness at the public level, a recent Polish experience was reported by Janowiak-Majeranowska et al. [25], in which they found that exploiting social media and organizing national days that were designed to improve public knowledge about HSCT seemed promising. These ideas seem doable and can also be applied to our community in Saudi Arabia.

Despite it being one of the few studies in Saudi Arabia that measure awareness and acceptance of HSCT as a cure for SCD, this study has many limitations. It was done via an online questionnaire that relies on the investigator and data collectors’ networks and may have led to a non-response bias due to the barrier of Internet accessibility in some areas and among certain types of populations in Jazan. In addition, the questionnaire failed to include a question related to national registry program enrollment. Further, with such methodology, we would not be able to confirm disease severity that is utmost in need for HSCT. Yet we believe that we included real regional information of the public in Jazan province.

## Conclusions

To conclude, this study was conducted to fill the gap in knowledge about public awareness and attitude toward HSCT for SCD, as most previous studies in Saudi Arabia were directed to medical students. We found out that almost 57% of the study participants were able to define HSCT. Most of the questions related to HSCT were answered correctly by those who have heard about HSCT. Further, only 42% of our study participants are willing to donate, a percent must be increased to match the real need for HSCT. National education programs must be directed to medical schools as well as the general population to increase awareness about HSCT, which may lead to a better attitude and willingness for HSCT donation.
